# Diet in 1-year-old farm and control children and allergy development: results from the FARMFLORA birth cohort

**DOI:** 10.3402/fnr.v60.32721

**Published:** 2016-08-16

**Authors:** Karin Jonsson, My Green, Malin Barman, Agneta Sjöberg, Hilde K. Brekke, Agnes E. Wold, Ann-Sofie Sandberg

**Affiliations:** 1 Division of Food and Nutrition Science, Department of Biology and Biological Engineering, Chalmers University of Technology, Gothenburg, Sweden; 2 Department of Internal Medicine and Clinical Nutrition, University of Gothenburg, Gothenburg, Sweden; 3 Department of Food and Nutrition, and Sport Science, University of Gothenburg, Gothenburg, Sweden; 4 Department of Nutrition, Institute of Basic Medical Sciences, University of Oslo, Oslo, Norway; 5 Clinical Bacteriology Section, Department of Infectious Diseases, University of Gothenburg, Gothenburg, Sweden

**Keywords:** atopy, dairy farm, fatty acids, dietary patterns, infants

## Abstract

**Background:**

A farming environment confers strong protection against allergy development. We have previously shown that farming mothers consume more full-fat dairy than control mothers, who instead consume more low-fat dairy, margarine, and oils; margarine and oil intake was associated with increased risk of allergy development in their children.

**Objectives:**

The aims of this study were to investigate the differences in diet between children in farming and control families at 1 year of age, to investigate the relation between the diets of the mothers and their children, and to relate the children's diet to allergy development.

**Design:**

The diet of 1-year-old children from dairy farming families (*n*=28) and from control families in the same rural area (*n*=37) was assessed by 24-h dietary recalls, followed by 24-h food diaries. Allergy was diagnosed by pediatricians at 3 years of age using strict predefined criteria.

**Results:**

Farm children had a higher intake of farm milk, whole cream, cholesterol, saturated fat, and fat in total and tended to eat more butter, while controls consumed more carbohydrates and poultry and tended to eat more margarine. Farm children also had higher intakes of homemade porridge/gruel, oily fish, and iodine. The intake of butter and whole milk in children and mothers correlated significantly in farm families but not in controls. A weak negative association was found between seafood intake and allergy development, while allergy was positively associated with the intake of pork as well as zinc in the control group; these intakes also correlated with each other.

**Conclusions:**

Consistent with mothers in farming families, the children consumed more full-fat dairy and saturated fat than did controls, but this could not be linked to the low risk of allergy in the farming group. Seafood intake might protect against allergy development, in accordance with earlier findings.

The incidence of allergy has increased during the last decades in countries with a Western lifestyle. A reduced exposure to microbes has been proposed to explain the increase (1, 2), but changes in diet may also contribute. For example, an increased consumption of omega-6 fatty acids has paralleled the increase in allergy development, (3) and consumption of margarine has been associated with an increased allergy prevalence (4, 5) Another common finding is that the consumption of fish by the mother and the early introduction of fish in the diet of the child is associated with protection from the development of allergy (6, 7).

Children who grow up on farms have considerably fewer allergies than those who grow up in the countryside, but not on a farm (8). Contact with livestock (9, 10), cattle feed (11), and the consumption of unpasteurized farm milk (9, 12) have been identified as independent protective factors. We have previously shown in the FARMFLORA birth cohort that farm mothers consume more full-fat milk and butter than do non-farm mothers living in rural areas, who instead consume more margarine and oils (13). Accordingly, the breast milk of farm mothers is richer in saturated fats and poorer in unsaturated fats (13). Furthermore, the consumption of margarine and oils by the mother was associated with an increased risk of allergy development in the offspring (13). Although fish consumption did not differ notably between the farm and control mothers in our cohort (13), we found that the proportions of the long-chain omega-3 (n-3) polyunsaturated fatty acid (PUFA) eicosapentaenoic acid (EPA) in serum phospholipids in 4-month-old infants were associated with less allergy development; serum EPA correlated with the breast milk proportions of EPA, which in turn correlated with the maternal consumption of oily fish during lactation (unpublished observations).

The aim of this study was to investigate whether the diet at 1 year of age differed between children raised on small dairy farms and control children in rural non-farming families, and whether the children's diet resembled those of their mothers. A second aim was to examine the relation between the diet at 1 year of age and subsequent allergy development.

## Methods

### Subjects

The FARMFLORA birth cohort includes 65 children from the Skaraborg County in the Southwestern part of Sweden, 28 of whom are being raised on small family-owned dairy farms, while 37 are controls living in the same rural area but not on farms, all included in this study. The recruitment of the pregnant women took place at maternity clinics between September 2005 and May 2008. Children who were born within the span of gestational weeks 36–42 were included in the study. Written consent was obtained from both parents and the study was approved by the Regional Ethics Committee in Gothenburg (No. 363-05).

### Assessment of diet in 1-year-old children

At the age of 1 year (10–14 months), the parents of the children were asked to complete a 24-h dietary recall followed by a 24-h food diary of their children's diet. The 24-h recalls were performed unannounced and followed a pre-decided interview protocol based on the five-step multiple-pass method developed by the United States Department of Agriculture (14). The protocol included questions about 1) all foods and beverages consumed during the last 24 h, 2) foods that are often forgotten, 3) when and where the foods and/or beverages were consumed, and 4) additional information, such as the amounts and brands, for all the reported foods and beverages. To shorten the interview, the fifth step of the original protocol was omitted, that is, the reproduction by the interviewer of all recorded food items, giving the interviewee a chance to remember any forgotten foods consumed by the child. The parents were instructed to initiate the 24-h food diary immediately after the interview was made, including registration of the amount and brand of the foods, at what time the foods were consumed, and how the foods were prepared. Weights were recorded aided by household scales or estimated by volume with household measures.

The collected dietary information was registered and calculated based on the food composition database of the Swedish National Food Agency using the software Dietist Net Pro (Kost och Näringsdata, version 15.11.02, Stockholm, Sweden).

No data of the children's weights were collected at 1 year of age. Instead, a reference weight of Swedish 1-year-old children (10.25 kg) (15) was used to obtain a reference value of the recommended energy intake, based on current nutrient recommendations (0.33 MJ/kg, mean for boys and girls) (16).

### Clinical examination

At the ages of 1.5 and 3 years, the children were examined by pediatricians to diagnose allergic rhinoconjuctivitis, asthma, and/or food allergies according to predefined protocols (17). Allergic rhinoconjuctivitis was defined as nose or eye symptoms after exposure to pollen or animal dander in combination with an allergen-specific IgE test to the correspondent inhalant allergen (Phadiatope, Phadia, Uppsala, Sweden). Asthma was diagnosed based on either persistent wheezing for ≥4 weeks, or ≥3 wheezing episodes combined with either: 1) eczema, allergic rhinoconjuctivitis, or food allergy; or 2) a response to leukotriene antagonists or inhaled glucocorticoids. For asthma diagnosis at 3 years of age, at least one wheezing episode had to have occurred after 2 years of age. Food allergy and sensitization against common food and inhalant allergens were assessed by blood tests (Phadiatope, Phadia, Uppsala, Sweden). Food allergy was defined based on immediate or late-onset reactions that rapidly improved after allergen elimination. The diagnosis was affirmed by a positive six-mix food test and/or an open-food challenge test. This was then followed by an Immunocap test (Phadia) for the identification of the relevant allergen. Atopic eczema was diagnosed according to the criteria by Williams (18) or Hanifin and Rajka (19). For the diagnosis of atopic eczema at the age of 3 years, symptoms had to be present after 2 years of age.

### Statistics

#### Multivariate analysis

Orthogonal Projections to Latent Structures with Discriminant Analysis (OPLS-DA) (20) was used to visualize the differences in the dietary patterns of farm and control children. Data were scaled using UV-scaling, and model validation was performed by cross-validation. Error bars denote the 95% confidence level, which was computed by jackknifing (the estimation of bias and variance based on subsets of the available data).

#### Univariable analysis

Due to a non-normal distribution of variables, a non-parametric Mann–Whitney *U* test was used for the evaluation of differences in dietary intake between farmers and controls and healthy and allergic children. For categorical variables, a χ^2^-test or Fishers’ exact test was used. Correlations were made using Spearman's *rho*. Two-tailed *P*-values <0.05 were used as the limit of significance. The univariable analyses were performed using SPSS (version 22, IBM Corporation New York, USA).

#### Multivariable analysis

Logistic regression was used for multivariable analysis (SPSS, version 22). Variables were included as covariates in adjusted models if *P*≤0.2 in a univariable analysis of healthy versus allergic children.

## Results

Characteristics of farm (*n*=28) and control (*n*=37) children are shown in Table 1. Farm and control children differed according to gender, with more male infants being born in the control group while farm fathers were less often allergic and had less education than control fathers.

**Table 1:** Characteristics of farm versus control children and healthy versus subsequent allergic children

	Farmers	Controls		Healthy	Allergic	
				
Variables	(*n*=28)	(*n*=37)	*P*^a^	(*n*=44)	(*n*=11)	*P*^a^
Antenatal characteristics						
Heredity^b^						
Mothers	7 (25%)	11 (30%)	0.68	9 (21%)	5 (46%)	0.12
Fathers	1 (4%)	12 (32%)	0.01	7 (16%)	3 (27%)	0.40
Maternal age at delivery, year	33 (21–42)	32 (22–41)	0.46^c^	32 (21–42)	34 (22–41)	0.71^c^
Education, level^d^ (1=lowest, 5=highest)						
Mothers	2 (1–5)	4 (1–5)	0.20^c^	4 (1–5)	3 (1–5)	0.78^c^
Fathers	2 (1–5)	2 (1–5)	0.02^c^	2 (1–5)	2 (2–4)	0.51^c^
Smoking during last month of pregnancy						
Mothers	0 (0%)	1 (3%)	1.00	1 (2%)	0 (0%)	1.00
Fathers	1 (4%)	4 (11%)	0.38	2 (5%)	2 (18%)	0.18
Cats or dogs in house at recruitment	21 (75%)	19 (51%)	0.05	31 (71%)	5 (46%)	0.16
Siblings	18 (64%)	17 (46%)	0.15	23 (52%)	6 (55%)	0.89
Birth characteristics						
Gestational week^e^	40 (37–42)	39 (36–42)	0.13^c^	39 (36–42)	39 (36–41)	0.14^c^
Cesarean section	3 (11%)	7 (19%)	0.50	5 (11%)	4 (36%)	0.07
Birth weight^f^, g	(2,780–4,740)	(2,440–4,830)	0.78^c^	(2,440–4,740)	(2,730–4,830)	0.14^c^
Infant characteristics						
Male gender	10 (36%)	23 (62%)	0.04	22 (50%)	9 (82%)	0.09
Maternal fish oil intake, 4 months postpartum	2 (11%)	0 (0%)	0.18	2 (7%)	0 (0%)	1.00
Intake of supplements of vitamin A+D at 1 year of age	17 (61%)	22 (60%)	0.92	30 (68%)	6 (55%)	0.49
Allergic at 3 years of age	1 (4%)	10 (32%)	0.02			

Data are presented as *n* (%) or medians (ranges).

a χ^2^-test (or Fishers’ exact test).

b Doctor's diagnosed asthma, rhinitis, or atopic eczema.

c Mann–Whitney *U* test.

d 1=Elementary school, 2=upper secondary school 2–3 years or equivalent, 3=qualified graduate from upper secondary engineering course, 4=university <1 year, 5=university >1 year.

e *n*=27 farmers and 36 non-farmers, and *n*=43 healthy and 10 allergic subjects, respectively.

f *n*=36 non-farmers, and *n*=10 allergic subjects.

At 3 years of age, 10 children in the control group and one in the farming group were allergic. In the allergic group, cesarean section tended to be more prevalent and male gender tended to be higher compared to the healthy group.

### Energy intake

All 65 children had either the 24-h dietary recall and/or the 24-h food diary completed; 74% completed both the recall and diary, 14% completed only the dietary recall and 12% only the food diary, with no difference between farming and control families or between families with healthy or allergic children. The median energy intake of all children was 4,202 kJ from foods, excluding breast milk, and did not differ significantly between farm and control children or healthy and subsequently allergic children. The children had a median energy intake per body weight of 0.41 MJ/kg, which exceeded the current recommendations of 0.33 MJ/kg (16). Five farm children and four control children were partially breastfed at 1 year of age (*P*=0.10). Of these, one child (in the farm group) received substantial amounts of breast milk at this age and consumed only 712 kJ per day from foods other than breast milk. The duration of exclusive breastfeeding tended to be longer in farm children compared to control children (4 vs. 3.5 months, *P*=0.11), although the duration of any breastfeeding did not differ (8 months in both groups, *P*=0.74).

### Diet in farm and control children

A broad variety of foods in the children's diets were reported. Food items consumed by less than 25% of the children were not included in the tables and figures; such items were olive oil, low-fat cream, lean fish, shellfish, game/lamb, nuts/seeds, peanuts, soft drinks, mashed potatoes from powder, muesli/granola, coconut milk, vegetable based milk, mushrooms, ketchup/chili sauce, whey cheese, and mayonnaise dressing.

Differences between farm and control children in the intake of food groups or food items, macronutrients and fatty acids, and micronutrients were analyzed as both absolute and energy-adjusted values (Tables 2–4). In Table 2, the median intakes of different food items and food groups are displayed. Farm children consumed more farm milk (*P*<0.001), whole cream (*P*=0.02), oily fish (*P*=0.02), and homemade porridge/gruel (*P*=0.05) than did controls, while the latter group consumed more poultry (*P*=0.03; Table 2). Children in farming families tended to eat more butter (*P*=0.08), while controls tended to consume more margarine (*P*=0.06; Table 2). Significances were similar for energy-adjusted values (Table 2).

**Table 2:** Absolute and energy-adjusted intakes of food items/groups in farm and control children at 1 year of age

	Absolute values (g)			Energy-adjusted values (g/MJ)		
				
	Farmers	Controls		Farmers	Controls	
				
Food items/groups	(*n*=28)	(*n*=37)	*p*^a^	(*n*=28)	(*n*=37)	*p*^a^
Butter^b^	3 (0–10)	0 (0–5)	0.12	1 (0–3)	0 (0–1)	0.08
Margarine	0 (0–3)	2 (0–6)	0.07	0 (0–1)	0 (0–1)	0.06
Rapseed oil	1 (0–3)	1 (0.5–4)	0.77	0 (0–1)	0 (0–1)	0.98
Other oils	0 (0–1)	0 (0–01)	0.84	0 (0–0)	0 (0–0)	0.95
Oils total	2 (0–4)	2 (1–4)	0.90	1 (0–1)	1 (0–1)	0.82
Margarine and oil	3 (1–6)	5 (2–9)	0.10	1 (0–2)	1 (1–2)	0.19
Milk products (≤1.5% fat)^c^	0 (0–30)	15 (0–80)	0.10	0 (0–9)	4 (0–20)	0.13
Milk products (>1.5% fat)^c^,^d^	58 (9–120)	25 (0–75)	0.17	15 (3–26)	7 (0–19)	0.12
Farm milk^e^	0 (0–75)	0 (0–0)	0.00	0 (0–21)	0 (0–0)	0.00
Milk total	77 (30–160)	100 (4–180)	0.93	20 (9–34)	27 (1–39)	1.00
Cream (27–41% fat)	1 (0–6)	0 (0–1)	0.02	0 (0–1)	0 (0–0)	0.02
Cheese	7 (0–19)	3 (0–12)	0.46	2 (0–4)	1 (0–3)	0.28
Oily fish	0 (0–12)	0 (0–0)	0.02	0 (0–4)	0 (0–0)	0.02
Seafood total	0 (0–12)	0 (0–3)	0.08	0 (0–4)	0 (0–1)	0.07
Pork^f^	8 (1–20)	10 (6–14)	0.83	2 (0–5)	2 (0–3)	0.68
Beef^f^	9 (3–22)	10 (6–15)	0.94	3 (1–6)	3 (1–4)	0.53
Processed meat	4 (0–25)	0 (0–18)	0.44	1 (0–6)	0 (0–4)	0.36
Poultry	0 (0–0)	0 (0–5)	0.03	0 (0–0)	0 (0–1)	0.03
Eggs	0 (0–9)	0 (0–1)	0.74	0 (0–2)	0 (0–0)	0.71
Porridge/gruel, homemade^g^	0 (0–43)	0 (0–0)	0.06	0 (0–8)	0 (0–0)	0.05
Porridge/gruel, from store^g^	76 (15–190)	110 (55–200)	0.36	22 (10–48)	30 (13–51)	0.50
Bread/rice/pasta/couscous	41 (15–58)	46 (15–77)	0.40	10 (5–18)	12 (4–16)	0.80
Crackers (non-sweetened)	0 (0–3)	0 (0–4)	0.66	0 (0–1)	0 (0–0)	0.71
Whole grain^h^	0 (0–0)	0 (0–0)	0.51	0 (0–3)	0 (0–0)	0.56
Grain foods, total^i^	46 (26–77)	60 (29–98)	0.47	13 (7–20)	13 (7–20)	0.88
Potatoes	38 (3–51)	30 (8–64)	0.99	9 (1–16)	9 (2–14)	0.76
Mashed potatoes	38 (7–51)	35 (8–65)	0.96	9 (3–16)	9 (2–16)	0.73
Root vegetables^j^	10 (0–22)	10 (0–30)	0.40	2 (0–6)	2 (0–6)	0.61
Vegetables^k^	30 (15–57)	42 (12–69)	0.40	6 (3–14)	10 (4–17)	0.55
Fruits/berries	68 (46–150)	84 (53–130)	0.67	17 (11–29)	21 (12–28)	0.90
Fruits and vegetables, sum^l^	150 (77–200)	18 (90–240)	0.22	33 (19–44)	37 (21–50)	0.46
Fruit juice	0 (0–25)	3 (0–25)	0.81	0 (0–8)	1 (0–7)	0.91
Sugar-rich foods^m^	31 (0–140)	44 (6–130)	0.82	10 (0–30)	16 (1–27)	0.99

Data are presented as medians (interquartile ranges).

a Mann–Whitney *U* test.

b Includes butter-based spreads.

c Includes sour milk products.

d Includes farm milk.

e Incudes both pasteurized and unpateurized farm milk.

f Includes processed meat.

g Wet weight.

h For bread: >5% fiber, for pasta: >50% whole grain.

i Bread, rice, pasta, crackers, cereals/granola, including whole grain.

j Excludes potatoes.

k Includes green peas, no other legumes.

l Root vegetables, vegetables, fruits and berries.

m Pastry, cake, candy, ice cream, fruit yoghurt, fool, and so on.

**Table 3:** Absolute and energy-adjusted intakes of macro nutrients and fatty acids in farm and control children at 1 year of age

	Absolute values (g)			Energy-adjusted values (g/MJ)		
				
	Farmers	Controls		Farmers	Controls	
				
Energy and nutrients	(*n*=28)	(*n*=37)	*p*^a^	(*n*=28)	(*n*=37)	*p*^a^
Energy and macronutrients						
Energy, kJ	4,009 (3,383–5,093)	4,247 (3,755–4,833)	0.62			
Energy, kcal	958 (809–1,217)	1,011 (897–1,154)	0.62			
Protein, g	35 (26–43)	34 (30–43)	0.69	8.2 (7.6–9.2)	8.6 (7.7–9.2)	0.54
Carbohydrates, g	130 (110–140)	135 (124–151)	0.11	31 (29–32)	32 (30–34)	0.02
Fiber, g	11 (9–13)	11 (8–13)	0.93	2.6 (2–3.1)	2.5 (2.2–2.9)	0.60
Fat, g	33 (26–49)	32 (27–41)	0.70	8.6 (7.8–9.7)	7.6 (7–8.7)	0.01
Cholesterol, mg	85 (57–118)	60 (35–96)	0.07	22 (15–30)	17 (10–22)	0.02
Fatty acids						
Saturated fatty acids, g	14 (9–21)	13 (10–17)	0.47	3.6 (3–4.3)	3 (2.5–3.9)	0.02
4:0–10:0, g	1.0 (0.7–1.8)	0.6 (0.4–1.3)	0.06	0.26 (0.20–0.40)	0.15 (0.10–0.28)	0.01
12:0, g	0.6 (0.3–1.0)	0.5 (0.3–0.8)	0.54	0.16 (0.1–0.19)	0.12 (0.09–0.17)	0.28
14:0, g	1.4 (1.0–2.0)	1.0 (0.7–1.6)	0.06	0.39 (0.31–0.52)	0.25 (0.18–0.37)	0.002
16:0, g	7.8 (5.0–11)	7.6 (5.0–9.0)	0.42	2 (1.6–2.3)	1.8 (1.6–2)	0.07
18:0, g	2.3 (1.6–3.4)	2.0 (1.4–2.6)	0.25	0.6 (0.5–0.7)	0.5 (0.3–0.6)	0.02
20:0, g	0.08 (0.05–0.1)	0.07 (0.04–0.1)	0.80	0.02 (0.01–0.02)	0.02 (0.01–0.02)	0.37
Monounsaturated fatty acids, g	12 (9–17)	12 (9–14)	0.90	3 (2.6–3.4)	2.8 (2.5–3.1)	0.16
16:1, g	0.5 (0.3–0.6)	0.3 (0.2–0.4)	0.08	0.11 (0.07–0.16)	0.07 (0.05–0.11)	0.01
18:1, g	10 (9.0–16)	11 (9.0–13)	0.91	2.8 (2.4–3.2)	2.6 (2.3–2.9)	0.27
Poly unsaturated fatty acids, g	5 (4–6)	5 (4–7)	0.42	1.3 (1.2–1.5)	1.3 (1.1–1.5)	0.91
18:2, n-6, g	4.2 (3.3–4.6)	4.8 (3.4–5.4)	0.20	1 (0.89–1.15)	1 (0.91–1.20)	0.20
18:3, n-3, g	0.7 (0.5–0.9)	0.7 (0.5–1)	0.74	0.17 (0.13–0.22)	0.17 (0.11–0.23)	0.99
20:4, n-6, g	0.05 (0.02–0.07)	0.03 (0.02–0.05)	0.13	0.01 (0.01–0.02)	0.01 (0–0.01)	0.04
20:5, n-3 (EPA), g	0.01 (0.00–0.13)	0.01 (0.00–0.01)	0.19	0 (0–0.03)	0 (0–0)	0.24
22:5, n-3 (DPA), g	0.02 (0.00–0.07)	0.01 (0.01–0.03)	0.46	0 (0–0.02)	0 (0–0.01)	0.52
22:6, n-3 (DHA), g	0.04 (0.01–0.25)	0.02 (0.01–0.06)	0.34	0.01 (0–0.07)	0.01 (0–0.01)	0.35

EPA, eicosapentaenoic acid; DPA, docosapentaenoic acid; DHA, docosahexaenoic acid.

Data are presented as medians (interquartile ranges).

a Mann–Whitney *U* test.

**Table 4:** Intake of micronutrients in 1-year-old farm and control children

	Absolute values (weight)			Energy-adjusted values (weight/MJ)		
				
	Farmers	Controls		Farmers	Controls	
				
Nutrients	(*n*=28)	(*n*=37)	*P*^a^	(*n*=28)	(*n*=37)	*P*^a^
Vitamin A, RE	400 (250–660)	370 (280–600)	0.77	140 (100–180)	140 (99–200)	0.86
Vitamin B6, mg	1.0 (0.90–1.33)	1.1 (0.97–1.5)	0.18	0.27 (0.20–0.30)	0.26 (0.23–0.32)	0.31
Vitamin B12, µg	2.3 (1.8–3.8)	2.1 (1.5–2.8)	0.19	0.60 (0.40–0.80)	0.50 (0.40–0.70)	0.09
Vitamin C, mg	69 (54–87)	66 (48–95)	0.99	17 (14–20)	16 (12–22)	0.70
Vitamin D, µg	5.4 (3.7–7.4)	5.7 (3.2–7.7)	0.87	1.4 (0.90–2.0)	1.4 (0.70–2.0)	0.78
Vitamin E, mg	8.5 (6.3–11)	10 (6.9–16)	0.4	2.1 (1.7–2.8)	2.3 (1.6–3.0)	0.60
Calcium, mg	620 (460–790)	620 (480–770)	0.81	140 (110–180)	150 (110–180)	0.68
Folate, µg	120 (110–150)	130 (95–150)	0.58	32 (24–37)	29 (26–33)	0.24
Iodine, µg	38 (25–66)	33 (18–50)	0.18	10 (7.0–14)	7.0 (4.0–10)	0.03
Iron, mg	8.2 (6.7–9.5)	8.9 (5.8–11)	0.62	2.0 (1.6–2.5)	2.0 (1.5–2.9)	0.48
Magnesium, g	147 (132–171)	160 (130–170)	0.58	35 (30–40)	36 (32–39)	0.68
Niacin, NE	8.1 (6.0–9.1)	8.4 (7.0–9.5)	0.36	3.5 (3.1–3.7)	3.5 (3.0–4.0)	0.68
Phosphorus, mg	750 (540–920)	710 (580–860)	0.97	170 (150–190)	170 (150–190)	0.93
Potassium, g	1.5 (1.3–1.7)	1.5 (1.4–1.9)	0.55	0.37 (0.31–0.43)	0.36 (0.33–0.43)	0.64
Riboflavin, mg	1.0 (0.71–1.3)	0.95 (0.77–1.1)	0.68	0.24 (0.20–0.25)	0.23 (0.20–0.24)	0.75
Selenium, µg	13 (10–18)	13 (9.0–15)	0.45	3.0 (2.5–4.4)	2.9 (2.5–3.5)	0.25
Thiamine, mg	0.80 (0.68–0.95)	0.83 (0.69–1.1)	0.53	0.20 (0.16–0.23)	0.20 (0.17–0.24)	0.47
Zinc, mg	4.7 (3.8–6.1)	4.8 (3.9–5.6)	0.91	1.1 (1.0–1.3)	1.2 (1.0–1.2)	0.73

Data are presented as medians (interquartile ranges).

a Mann–Whitney *U* test.

**Fig. 1:**
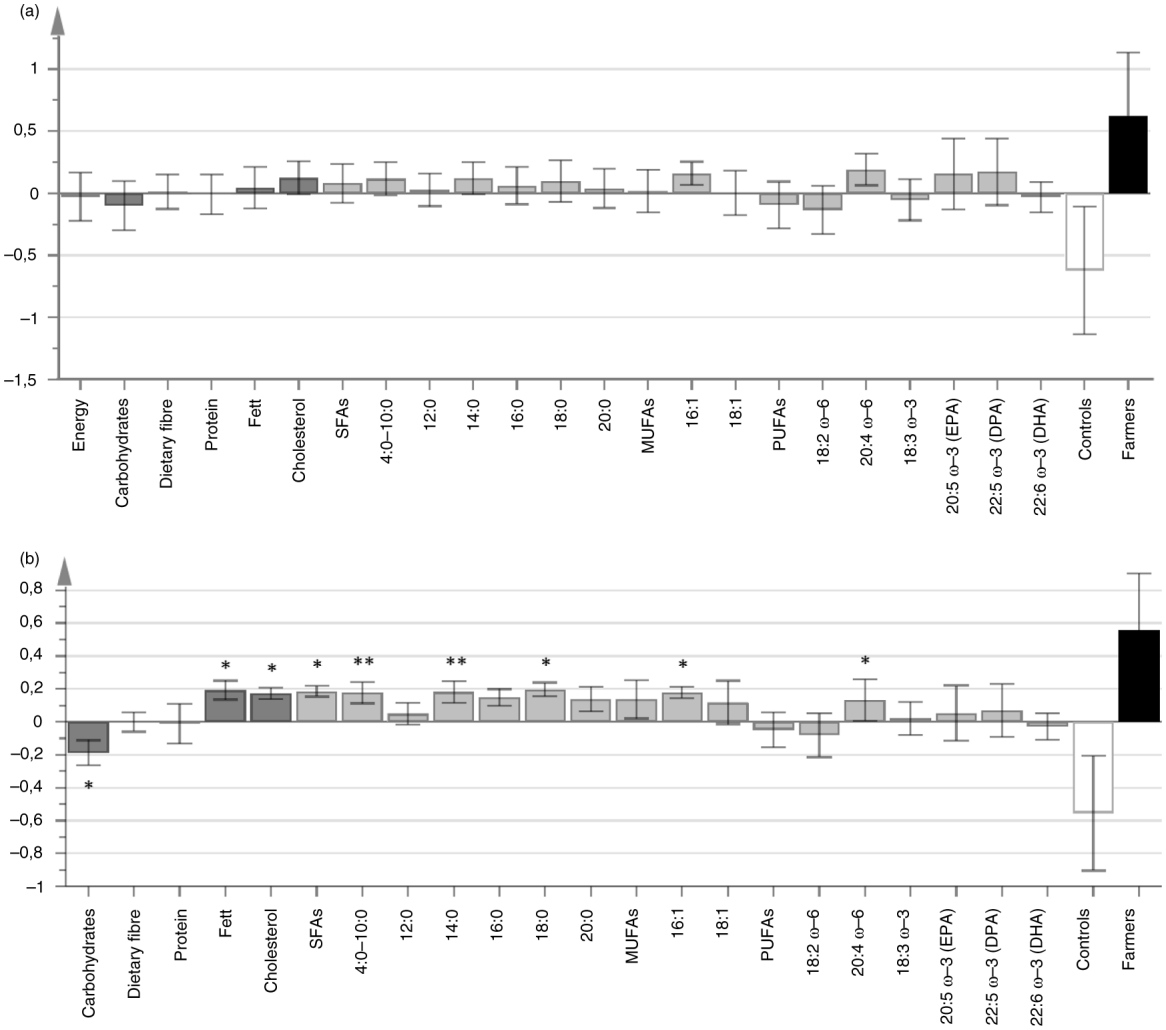
Loadings plot of OPLS-DA displaying differences in absolute (a) and energy-adjusted (b) intakes of energy, macronutrients, and fatty acids in farm and control children at 1 year of age. Variables pointing in the same direction as farm children are higher in farm children and vice versa. Error bars denote the 95% confidence level, calculated using jackknifing during cross-validation. The models contain two components; R^2^X=0.13 (a) and 0.37 (b), and Q^2^=0.06 (a) and 0.08 (b). Information about univariable significances has been added to the figure in retrospect, analyzed by univariable Mann–Whitney *U* test (*P≤0.05; **P≤0.01). *n*=28 farm children and 37 controls. *Abbreviations:* DHA, docosahexaeonic acid; DPA, docosapentaeonic acid; EPA, eicosapentaenoic acid; MUFAs, monounsaturated fatty acids; PUFAs, polyunsaturated fatty acid; ω, omega; SFAs, saturated fatty acids.

To visualize the differences in the intake of macronutrients and fatty acids between farm and control children, OPLS-DA was performed on the absolute and energy-adjusted variables (Fig. 1a and b). The absolute and energy-adjusted intakes of macronutrients and fatty acids in farm and control children are also shown in Table 3, which displays the median intakes and interquartile ranges. The differences between farm and control children were more pronounced if their diets were calculated as energy-adjusted values rather than absolute values. Farm children consumed significantly more fat (*P*=0.01), cholesterol (*P*=0.02), the fatty acids 4:0–10:0 (*P*=0.01), 14:0 (*P*=0.02), 18:0 (*P*=0.02), saturated fatty acids in total (*P*=0.02), as well as the fatty acids 16:1 (*P*=0.01) and arachidonic acid (*P*=0.04) compared to controls, who instead consumed more carbohydrates (*P*=0.02; Table 3, energy-adjusted values).

The absolute and energy-adjusted intakes of micronutrients in farm and control children are shown in Table 4. No significant differences were found between the farm and control children, except for iodine, of which farm children had a higher intake (*P*=0.03; Table 4).

### Correlations between selected nutrients and food items in the children's diet

To examine the origin of the fatty acids that were consumed in different amounts of farm and control children, we correlated the absolute intakes of fatty acids and food items (Table 5). Butter, milk, cream, cheese, and pork were the main contributors of the dietary fatty acids 4:0–10:0, 14:0, 18:0, and 16:1, while oily fish was the main contributor of arachidonic acid (Table 5).

**Table 5:** Correlations between the intakes of fatty acids^a^ and food items in farm and control children at 1 year of age

	Fatty acids									
	
	4:0–10:0		14:0		18:0		16:1		20:4^b^	
					
Foods	*rho*^c^	*P*	*rho*^c^	*P*	*rho*^c^	*P*	*rho*^c^	*P*	*rho*^c^	*P*
Butter	0.67	<0.001	0.69	<0.001	0.56	<0.001	0.56	<0.001	0.29	0.02
Milk	0.32	0.01	0.31	0.01	0.35	0.01	0.27	0.03	0.08	0.52
Cream	0.31	0.01	0.34	0.01	0.28	0.03	0.32	0.01	0.30	0.02
Cheese	0.58	<0.001	0.54	<0.001	0.46	<0.001	0.50	<0.001	0.38	0.01
Pork	0.46	<0.001	0.42	0.001	0.58	<0.001	0.56	<0.001	0.38	0.01
Beef	0.10	0.42	0.16	0.21	0.29	0.02	0.35	0.01	0.32	0.01
Oily fish	0.06	0.65	0.05	0.72	–0.8	0.55	0.23	0.08	0.43	<0.001

a Fatty acids, of which the intake differ significantly between farm and control children (absolute intakes were analyzed).

b Arachionic acid.

c Spearman's *rho*.

**Fig. 2:**
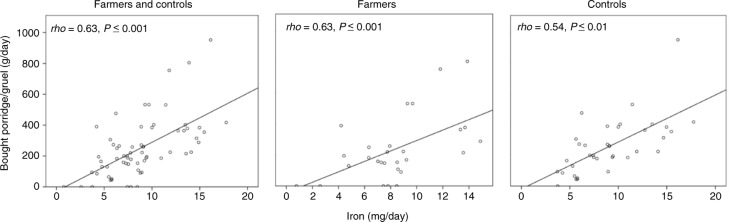
Correlations between the absolute intakes of iron and commercial porridge/gruel in 1-year-old farm and control children. Spearman's *rho* with two-sided significances of *P* ≤ 0.05 was used for the correlations.

Further, the correlations between the absolute intake of iron and different foods in the children's diet were analyzed to evaluate the main contributors of iron to their diet. The children's intake of bought porridge was the only food item or food group that correlated positively with the children's iron intake (*rho*=0.63, *P* ≤ 0.001, Fig. 2). Negative correlations to iron were found for grain foods (*rho*=−0.32, *P*=0.01), seafood (*rho*=−0.30, *P*=0.02), legumes (*rho*=−0.29, *P*=0.02), and charcuteries (*rho*=−0.26, *P*=0.04). When energy-adjusted intakes were analyzed, negative correlations were found also for butter (*rho*=−0.37, *P*=0.01), cheese (*rho*=−0.40, *P*=0.001), milk (*rho*=−0.27, *P*=0.03), pork (*rho*=−0.42, *P*=0.001), vegetables (*rho*=−0.34, *P*=0.01), and fruit (*rho*=−0.28, *P*=0.03).

### Correlations between the diets of mothers and children

**Fig. 3:**
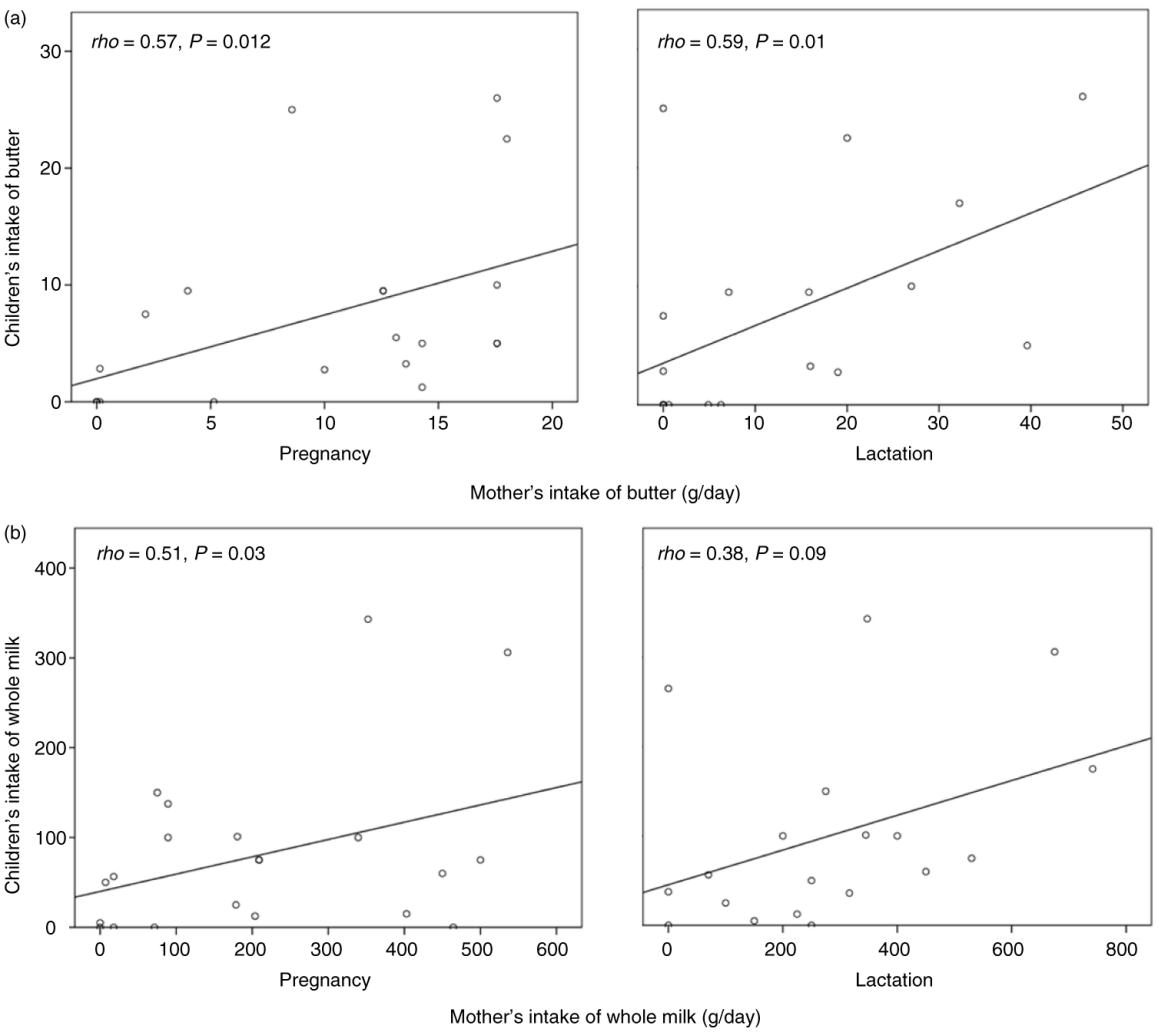
Correlations between butter (a) and whole milk (b) intake in children at 1 year of age and their mothers during pregnancy and lactation. The correlations were analyzed with Spearman's *rho* and two-sided significances of *P* ≤ 0.05. The children's diet and maternal diet during lactation (4 months postpartum) was assessed using 24-h dietary recalls followed by 24-h food diaries. The maternal diet during pregnancy was assessed using a semi-quantitative food frequency questionnaire.

Our hypothesis was that the diets of the pregnant and lactating mother and child living on a farm resemble each other and differ from those of non-farming families. Correlations were made between the maternal and child intake of foods that differed significantly between farm and control families, either in the children's or the mothers’ diets. Absolute intakes of the children's diet were used for correlations to the maternal intakes, as they were previously reported as absolute values (13). The farm children's intake of butter correlated with their mothers’ intake of butter both during pregnancy (*rho*=0.57, *P*=0.012) and lactation (*rho*=0.59, *P*=0.01; Fig. 3a), and the intake of whole milk correlated with maternal intake of whole milk during pregnancy (*rho*=0.51, *P* =0.03) and tended to correlate with the intake also during lactation (*rho*=0.38, *P*=0.09; Fig. 3b). The intake or whole cream in the farm children correlated with the mothers’ intake of whole milk (*rho*=0.47, *P*=0.04), but not whole cream, during pregnancy and tended to correlate with the maternal intake of whole cream during lactation (*rho*=0.40, *P*=0.08). The farm children's intake of margarine correlated with their mothers’ intake of margarine during lactation (*rho*=0.47, *P*=0.03); the intakes of oily fish did not correlate at all. In the control group, no significant correlations were found between the children's intake of full-fat milk and cream, butter, margarine, and oily fish and their mothers’ intake of these foods. Thus, the farming families were characterized by a common diet rich in full-fat dairy products, while no ‘family pattern’ could be detected in the control families.

### Diet in healthy and allergic children

One farm child (4%) and 10 control children (32%) were diagnosed as allergic by the age of 3 years (Table 1). The allergic farm child had eczema, along with six children from the control group (11% in total of the 11 allergic children). Of the eczematous control children, one had a concomitant food allergy and another had asthma. In total, four children (6%) had asthma, two (3%) had food allergy, and one (2%) allergic rhinitis. A shorter duration of any (6 vs. 8 months, *P*=0.02) and exclusive (0.5 vs. 4 months, *P*=0.01) breastfeeding was observed in the allergic group compared to the healthy group, although no significant differences were found for the rate of breastfeeding at 1 year of age (9% vs. 18%, *P*=0.13). Significant differences in the diets of healthy and allergic children are displayed in Table 6. Healthy children consumed more seafood (*P*=0.02) and tended to eat less pork (*P*=0.052; Table 6). Since only one farm child developed allergy, analyses were made also in control children only; the higher intake of seafood in healthy infants became a trend (*P*=0.14), while the lower intake of pork became significant (*P*=0.02; Table 6). Additionally, a higher intake of zinc was observed in allergic control children (*P*=0.04; Table 6). Crude and adjusted odds ratios for seafood and pork are displayed in Tables 7 and 8, respectively. We have observed that the proportions of the long-chain n-3 PUFA EPA in children's sera at 4 months of age were associated with protection from allergy (unpublished observations), while the maternal intake of margarine and oils during pregnancy and lactation were positively associated with allergy development (13). Therefore, we investigated whether the children's intake of pork or seafood correlated with serum EPA proportions and the maternal intake of margarine and oils. The intake of seafood at 1 year of age was found to correlate with EPA in infant sera at 4 months (*rho*=0.35, *P*=0.01). Similar results were obtained when energy-adjusted values were used in the calculation (*rho*=0.34, *P*=0.02); no correlation was found between seafood and the maternal intake of margarine and oils. Also, no correlations were found between pork and serum EPA or margarine and oil intake.

**Table 6:** Food items/groups of which the intake differs between healthy and subsequent allergic children; in the entire cohort and controls only

	Absolute values (g)			Energy-adjusted values (g/MJ)		
				
Foods and nutrients	Healthy (*n*=44)	Allergic (*n*=11)	*P*^a^	Healthy (*n*=44)	Allergic (*n*=11)	*P*^a^
Farmers and controls						
Seafood, g	0 (0–10)	0 (0–0)	0.02	0 (0–3)	0 (0–0)	0.02
Pork, g	7 (0–15)	10 (7–30)	0.08	2 (0–4)	2 (2–7)	0.052
Zinc, mg	5.0 (4.0–6.0)	5.0 (4.0–7.0)	0.11	1.1 (1.0–1.2)	1.3 (1.1–1.4)	0.73
				
	Healthy (*n*=21)	Allergic (*n*=10)	*P*	Healthy (*n*=21)	Allergic (*n*=10)	*P*
	
Controls						
Seafood, g	0 (0–9)	0 (0–0)	0.14	0 (0–3)	0 (0–0)	0.14
Pork, g	4 (0–12)	12 (7–30)	0.053	1 (0–3)	3 (2–7)	0.02
Zinc, mg	5.0 (4.0–5.0)	5.0 (5.0–7.0)	0.08	1.2 (1.1–1.2)	1.3 (1.1–1.4)	0.04

Data are presented as medians (interquartile ranges).

Allergy was diagnosed at 3 years of age.

a Mann–Whitney *U* test.

**Table 7:** Logistic regression models of intake of seafood at 1 year of age and allergy in the child at 3 year of age

	Farmers and controls			Controls		
		
	Healthy (*n*=44)			Healthy (*n*=21)		
		
	Allergic (*n*=11)			Allergic (*n*=10)		
		
Predictor variables	OR	95% CI	*P*	OR	95% CI	*P*
Seafood alone	0.41^a^	0.14–1.26	0.12^b^	0.45	0.14–1.43	0.17
Model 1						
Seafood	0.50	0.17–1.48	0.21	0.52	0.14–1.89	0.32
Length of exclusive breastfeeding, months	0.63	0.42–0.96	0.03	0.58	0.34–0.99	0.05
Model 2						
Seafood	0.43	0.14–1.33	0.14	0.39	0.11–1.44	0.16
Length of partial breastfeeding, months	0.84	0.69–1.02	0.08	0.81	0.63–1.03	0.08
Model 3						
Seafood	0.35	0.11–1.12	0.08	0.31	0.08–1.30	0.11
Cesarean section	8.43	1.2–59.2	0.03	11.7	0.89–1.54	0.06
Model 4						
Seafood	0.43	0.14–1.29	0.13	0.42	0.12–1.45	0.17
Female gender	0.21	0.04–1.16	0.07	0.15	0.02–1.50	0.11
Model 5						
Seafood	0.47	0.26–0.83	0.01	0.47	0.14–1.52	0.20
Maternal heredity	3.14	0.71–13.9	0.13	3.43	0.61–19.2	0.16
Model 6						
Seafood	0.41	0.13–1.29	0.13	0.48	0.14–1.66	0.25
Gestational week^c^	3.14	0.71–13.9	0.13	0.74	0.46–1.21	0.23
Model 7						
Seafood	0.44	0.15–1.33	0.15	0.46	0.14–1.54	0.21
Birth weight^d^, g	1.00	0.99–1.00	0.58	1.0	0.99–1.00	0.85
Model 8						
Seafood	0.39	0.12–1.27	0.18	0.44	0.14–1.38	0.16
Cats or dogs in house at recruitment	0.34	0.08–1.43	0.14	0.37	0.07–1.92	0.24
Model 9						
Seafood	0.44	0.15–1.31	0.14	0.46	0.14–1.48	0.19
Paternal smoking last month of pregnancy	2.68	0.32–22.2	0.36	1.47	0.17–12.9	0.73

Odds ratios (ORs) and confidence intervals (CIs) for every g/MJ/d of seafood intake at 1 year of age and allergy at 3 years of age.

Variables with *p*≤0.2 in univariable analysis are included as covariates in separate models.

a OR=0.03 for every 5 g/MJ/d.

b *P*=0.01 in likelihood ratio testing (χ^2^).

c One healthy farmer and one allergic control child missing.

d One allergic control child missing.

**Table 8:** Logistic regression models of intake of pork at 1 year of age and allergy in the child at 3 years of age

	Farmers and controls			Controls		
		
	Healthy (*n*=44)			Healthy (*n*=21)		
		
	Allergic (*n*=11)			Allergic (*n*=10)		
		
Predictor variables	OR	95% CI	*P*	OR	95% CI	*P*
Pork alone	1.28	1.02–1.6	0.03	1.37^a^	1.01–1.84	0.04
Model 1						
Pork	1.39	1.01–1.92	0.04	1.70	1.03–2.81	0.04
Length of exclusive breast feeding, months	0.53	0.34–0.84	0.01	0.43	0.21–0.86	0.02
Model 2						
Pork	1.39	1.03–1.87	0.03	1.56	1.06–2.30	0.03
Length of partial breast feeding, months	0.77	0.62–0.97	0.03	0.74	0.54–0.99	0.05
Model 3						
Pork	1.25	0.98–1.59	0.07	1.33	0.98–1.80	0.07
Cesarean section	3.28	0.62–17.5	0.16	2.18	0.3–15.9	0.44
Model 4						
Pork	1.34	1.03–1.76	0.03	1.53	1.04–2.25	0.03
Female gender	0.17	0.03–1.04	0.06	0.07	0.00–1.55	0.09
Model 5						
Pork	1.28	1.02–1.6	0.03	1.40	1.01–1.94	0.04
Maternal heredity	3.33	0.76–14.7	0.11	5.67	0.85–37.6	0.07
Model 6						
Pork	1.20	0.93–1.58	0.16	1.36	0.98–1.88	0.06
Gestational week^b^	0.70	0.45–1.09	0.11	0.70	0.42–1.14	0.15
Model 7						
Pork	1.26	0.97–1.63	0.09	1.36	0.99–1.88	0.06
Birth weight^c^, g	1.00	1.00–1.00	0.29	0.99	0.99–1.00	0.50
Model 8						
Pork	1.25	0.98–1.58	0.07	1.35	0.99–1.82	0.06
Cats or dogs in house at recruitment	0.54	0.12–2.35	0.41	0.67	0.12–3.75	0.65
Model 9						
Pork	1.30	1.03–1.60	0.03	1.38	1.02–1.87	0.04
Paternal smoking last month of pregnancy	6.40	0.67–61.9	0.11	3.31	0.31–35.4	0.32

Odds ratios (ORs) and confidence intervals (CIs) for every g/MJ/d of pork intake at 1 year of age and allergy at 3 years of age.

Variables with *P*≤0.2 in univariable analysis are included as covariates in separate models.

a OR=4.8 for every 5 g/MJ/d.

b One healthy farmer and one allergic control child missing.

c One allergic control child missing.

## Discussion

We have previously shown that farm mothers in the FARMFLORA birth cohort consume more saturated fat and full-fat dairy products during pregnancy and lactation, while control mothers consume more margarine and oils (13). In this study, we show that similar differences between the diet of farm and non-farm families occur among 1-year-old children. Thus, children living on a dairy farm consumed significantly more full-fat cream and saturated fat and tended to consume more butter, whereas control children tended to consume more margarine. These results are in line with a cross-sectional study of 6–13-year-old Finnish farm children, who had a higher intake of farm milk, whole milk, and butter but less margarine (21). In our study, the farm children's intake of butter correlated with the mothers’ butter intake, and the children's intake of whole milk correlated with maternal whole milk intake, although less pronounced. No correlations of these foods between mother and child were observed in controls, probably resulting from their less frequent consumption of full-fat dairy products.

Despite the farm mothers’ higher intake of full-fat milk during lactation observed in our previous study (13), we could not find any significant differences in the consumption of full-fat or low-fat milk between the 1-year-old farm and control children (except for higher intakes of farm milk in farm children). This might be explained by national guidelines that advocate the restriction of milk consumption up to 1 year of age, which are based on concerns regarding the low iron content in milk. In accordance, the children's energy-adjusted milk intake correlated negatively with the energy-adjusted iron intake. Commercially bought porridge or gruel was the only food group or item for which the intake correlated positively with the intake of iron. In addition to milk, the intake of several other food groups correlated negatively with the iron intake; a high intake of foods like vegetables, grains, dairy, fish, and pork is likely to be an indicator of a lower intake of porridge/gruel, which is fortified with iron when bought commercially. Accordingly, negative correlations were found between the intake of bought porridge/gruel and the intake of most of the foods that correlated negatively with iron intake. However, restricting the intake of several food groups in favor of bought porridge/gruel might counteract the acceptance of a broad range of foods, which in turn may influence the future diversity of a child's diet negatively.

The children's intake of nutrients did not differ substantially between farm and control children. The majority of the children had intakes of vitamin D, iodine, and selenium that were considerably lower than current recommendations (10, 70, and 20 µg, respectively) (16). Although only significant for iodine, the children of farmers less often had a diet deficient in the above-mentioned micronutrients, vitamin D, iodine, and selenium, which is most likely explained by their significantly higher intake of oily fish; fish is a good source of all these three micronutrients. Ten farm children (36%) and four control children (11%) had fish during any of the 2 days of registration. However, the true intake of fish is difficult to capture using a dietary assessment of only 2 days, since fish is generally consumed at irregular intervals (22). For just above half of all the farm and control children, respectively, the intake of supplements of vitamin A+D was reported; hence, the true vitamin D intake was larger in both groups. No correlations were found between the intake of vitamin D supplementation and fish intake (data not shown). A higher intake of oily fish was not observed among the Finnish farm children compared to controls (21). Instead, the controls consumed more fish in total; hence our findings of a higher oily fish intake in farm children may not characterize farm children's diet in general.

The intake of seafood at 1 year of age was negatively associated with being allergic at 3 years of age. The early intake of fish has previously been associated with protection against allergy development (6, 7, 23, 24). Accordingly, we have previously shown that high proportions of the long-chain n-3 PUFA EPA in sera at 4 months of age were associated with a lower risk of developing allergy in the FARMFLORA cohort, although not related to farm residence (unpublished observations). The intake of seafood in the 1-year-olds correlated with the proportions of EPA in the sera at 4 months of age, which in turn was related to EPA in breast milk and maternal intake of oily fish during pregnancy. Also, the breast milk proportions of EPA correlated with maternal fish intake during lactation. Thus, the intake of fish in the mothers and the 1-year-old children were associated. Whether it is the maternal or infant fish intake that confers protection against allergy cannot thereby be determined. Unfortunately, no serum samples were obtained at 1 year of age; hence, we did not analyze EPA or other long-chain PUFAs in sera from the 1-year-olds.

In contrast to seafood, the intake of pork was positively associated with allergy development. No substantial decrease in the odds ratio for pork and allergy was observed when adjusted for potential covariates in logistic regression. One could suspect that the consumption of more pork may result in the consumption of less fish, but no correlations were found between the children's pork and fish intakes. However, the odds ratio for the association of pork intake and allergy was moderate (1.37), and there are no previous reports of pork consumption as a risk factor for allergy development; hence, the finding should be interpreted with caution. Further, the intake of zinc was higher in children who later developed allergy than in healthy children. Correlations were made in retrospect between pork intake and dietary zinc content, and we found that these were positively correlated. Hence, the association between pork and allergy may be mediated by zinc. However, although zinc has immunomodulatory properties and could play a role in allergy development, there are no previous reports of positive associations between zinc and allergy, to the best of our knowledge.

The dietary pattern of a higher intake of full-fat dairy, including farm milk, which characterized the farm children in our study, was not related to allergy protection. However, unpasteurized milk has been associated with a lower risk of allergy in a number of studies (9, 12). In our study, the intake of farm milk was reported for 13 farm children, six of whom consumed unpasteurized milk. No control family reported the consumption of unpasteurized milk in the children. As all allergy cases except one occurred in the control group, we cannot determine whether the consumption of unpasteurized milk contributed to the pronounced protection from allergy development in the farm children.

## Conclusions

We could show a similar dietary pattern in farm children and their mothers, including more full-fat dairy, fat in total, and saturated fatty acids compared to control infants, who instead tended to consume more margarine; this pattern was, however, not linked to the protective effect on allergy development of growing up on a farm. However, the farm children consumed more oily fish, and a weak negative association between seafood intake and allergy development was found; the pork intake and zinc content of the diet were instead correlated with one another and were positively associated with the risk of allergy development in the control infants.
